# Red Cell Distribution Width As a Predictor of Mortality in Patients With Sepsis

**DOI:** 10.7759/cureus.12912

**Published:** 2021-01-25

**Authors:** Vinai Krishna, Gopalakrishna Pillai, Sheejamol Velickakathu Sukumaran

**Affiliations:** 1 Internal Medicine, Amrita Institute of Medical Sciences and Research Centre, Kochi, IND; 2 Biostatistics, Amrita Institute of Medical Sciences and Research Centre, Kochi, IND

**Keywords:** red cell distribution width, sepsis, mortality

## Abstract

Introduction

Sepsis is a common medical condition that is associated with very high mortality and, in survivors, long-term morbidity. Conventional inflammatory markers, such as CRP, erythrocyte sedimentation rate (ESR), and white blood cell count (WBC), have proven to have a limited utility in sepsis patients by virtue of their poor sensitivity and specificity for infections. Recently, the commonly used index of red cell distribution width (RDW) has been implicated as a prognostic marker in sepsis. This study aimed at assessing the role of RDW in predicting outcomes such as mortality in patients with sepsis and to study its role as a prognostic marker.

Methods

This was conducted as a prospective comparative observational study over a two year period between 2018 and 2020 in a tertiary care center in South India. In total, 60 adult patients above 18 years of age who were diagnosed to have severe sepsis and septic shock were selected to be part of the study. To find the survival probability on overall survival, Kaplan-Meier analysis was used and a comparison was done using the Log-rank test. To find the most significant predictors of mortality, cox regression analysis was applied.

Results

A total of 60 patients (n=60) were enrolled in this study out of which 30 (50%) patients had a rise in RDW and 30 (50%) patients had a fall in RDW. A total of 17 (28.3%) patients died during this study. In the rise in RDW group, there were 12 (40%) deaths while in the fall in the RDW group, there were five (16.7%) deaths. There was a statistically significant association found between mortality with rise and fall in RDW (p<0.05). A cox multivariate regression analysis demonstrated statistically significant associations between a rise in RDW (p<0.05, HR: 5.6, CI 1.4 to 21.9) and serum hemoglobin < 13.6 g/dL (p<0.05, HR: 3.6, CI 1.1 to 11.4) with mortality in this study. Kaplan-Meier analysis of rise and fall in RDW with survival trended towards better survival outcomes in the fall in the RDW group but was not significant (p=0.07).

Conclusion

We found that an increase in RDW from baseline during the initial 72 hours after admission is significantly associated with adverse clinical outcomes including mortality. The mortality in the rise in the RDW group, as well as overall mortality, were significantly higher than the mortality in the reduced RDW group. Hence, serial RDW measurements could be used as a prognostic factor in severe sepsis and septic shock.

## Introduction

Sepsis is a common medical condition that is associated with very high mortality and, in survivors, long-term morbidity. The World Health Organization (WHO) has made sepsis a global health priority since 2017 and has resolved to improve the prevention and management of sepsis [1]. The Surviving Sepsis Campaign has emphasized the importance of the early diagnosis in the prognosis of sepsis, but the diagnosis of sepsis has been a difficult task [2]. This is because of the varying manifestations of sepsis, and the inconsistent nature of laboratory confirmation of infection. Numerous biomarkers have been studied for diagnosing sepsis. C-reactive protein (CRP), procalcitonin, presepsin, etc are examples of such markers that have been widely studied [3-5]. However, these conventional inflammatory markers such as CRP, erythrocyte sedimentation rate (ESR), and white blood cell count (WBC), have proven to have a limited utility in sepsis patients by virtue of their poor sensitivity and specificity for infections. In addition, looking at microbiological cultures which are considered the gold standard diagnostic method for sepsis, often do not reflect the patient response of systemic inflammatory response syndrome (SIRS) or the onset of multiple organ dysfunction syndrome (MODS) and is time-consuming. These disadvantages of cultures and available blood markers have led to research to find more sensitive and specific markers. More recently, the commonly used index of red cell distribution width (RDW) has been implicated as a prognostic marker in sepsis [6].

RDW is the variation in size of all the red blood cells (RBCs). It increases when there is an excess of reticulocytes in the circulation. In addition to its role in the diagnosis of anemia, RDW is an important prognostic marker in cardiovascular disorders, community-acquired pneumonia, pulmonary embolism, and critical illnesses [7]. Oxidative stress and inflammation are thought to reduce RBC survival as well as suppress maturation which results in the release of premature RBCs into the circulation, and subsequently contributing to elevated RDW. Oxidative stress and inflammation are also the essential components of the sepsis cascade [6]. Complete blood count (CBC) is routinely done in most suspected sepsis patients admitted, by automated analyzers worldwide. RDW is provided as a part of the CBC report by automated analyzers. CBC is inexpensive, widely available, and has a fast turn around time. Hence, RDW may have a clinical utility in identifying septic patients who have severe sepsis, and those who need aggressive therapy. RDW could also be useful as a prognostic tool in cases of severe sepsis as reported in recent literature [6-9]. Hence, this study was conducted to assess the role of RDW in predicting outcomes such as mortality in patients with sepsis and to study its role as a prognostic marker.

## Materials and methods

This was conducted as a prospective comparative observational study over a two-year period between 2018 and 2020 in a tertiary care center in South India. Patients with sepsis satisfying the inclusion criteria were selected from the intensive care unit (ICU), emergency department, and medical departments. In total, 60 patients were selected to be part of the study including 30 patients with a rise in RDW and 30 patients with falls in RDW. Adult patients above 18 years of age and who were diagnosed to have sepsis were included in the study. Patients were excluded from the study if they had received any blood or blood product transfusion in the previous week of admission, had undergone chemotherapy in the previous year, had a previous history of diseases affecting red cells or the bone marrow, were on drugs known to change the morphology of red blood cells, had a blood loss of more than 10% of the blood volume in the recent past, and patients in whom aggressive management had been withdrawn. 

Definitions and data collection

Sepsis has now been defined as a life-threatening organ dysfunction caused by a dysregulated host response to infection, which manifested as systemic inflammatory response syndrome (SIRS) to the causative infection [10]. SIRS was defined as--two or more of--temperature >38°C or <36°C, heart rate >90/min, respiratory rate >20/min or PaCo2 <32 mm Hg (4.3 kPa), white blood cell count >12,000/mm3 or <4000/mm3 or >10% immature bands [11]. 

Once a patient was identified as having sepsis according to the inclusion criteria, they were further scrutinized by narrowing them down by whether the treating physician had ordered a complete blood count as part of standard treatment care. Only patients in whom a complete blood count and RDW were ordered at the time of admission, and in whom an initial blood culture was positive were included in the study. Informed consent was taken. Blood was taken from the patient to analyze the serum RDW levels. 

RDW was measured as a part of automated CBC and the normal reference range for our institution was 11.6-14.8%. Baseline RDW at admission and a serial value at 72 hours were recorded. Baseline characteristics including demographic information and pre-existing chronic comorbidities were collected. Charlson Comorbidity Index (CCI) score was used to assess the burden of chronic disease. The Acute Physiology and Chronic Health Evaluation (APACHE) score and Sequential Organ Failure Assessment (SOFA) score were determined using worst values within the initial 24 hours of admission to assess the severity of the disease. SOFA score was calculated by parameters such as PaO2/FiO2, platelet count, bilirubin, blood pressure and use of the inotropic agent, Glasgow coma score scale, and creatinine or urine output. In addition, WBC count, hemoglobin level, and hematocrit were measured as part of automated CBC. 

Statistical analysis

Based on the incidence rate of mortality among patients with a rise in RDW (50.9%) and fall in the RDW (11.9%) observed in a previous publication, with 95% confidence, and 10% margin of error, the sample size was calculated to be 30 patients in each group [12]. To test the statistical significance of the difference in the proportion of mortality and other categorical factors between the two groups, the chi-square test was used. To test the statistical significance of the difference in mean of continuous variables between the two groups, student’s t-test was used. To find the survival probability on overall survival, Kaplan-Meier analysis was used and a comparison was done using the Log-rank test. To find the most significant predictors on mortality, a cox-regression analysis was applied.

## Results

A total of 60 patients (n=60) were enrolled in this study out of which 30 (50%) patients had a rise in RDW and 30 (50%) patients had a fall in RDW. Thirty-four patients (56.7%) were males while 26 (43.3%) were females. Seventeen males (56.7%) had a rise in RDW while 13 females (43.3%) had a rise in RDW. Seventeen males (56.7%) had a fall in RDW while 13 females (43.3%) had a fall in RDW. There was no statistically significant association between sex with rise or fall in RDW (p= 1). The ages ranged from 25 to 85 years with a mean ± standard deviation (M±SD) of 61.7 ± 15.5 years. In the rise in RDW group, the M±SD was 61.9 ± 17 years. In the fall in the RDW group, the M±SD was 61.6 ±14 years. There was no statistically significant association identified between age with rise or fall in RDW (p=0.941). 

The CCI scores ranged from zero to six with an M±SD of 1.7±1.65. In the rise in the RDW group, the M±SD was 1.7±1.5. In the fall in the RDW group, the M±SD was 1.7±1.8. There was no statistically significant association identified between CCI with rise or fall in RDW (p=1). The SOFA scores ranged from two to 20 with an M±SD of 10.1±4. In the rise in the RDW group, the M±SD was 9.7±3.1. In the fall in the RDW group, the M±SD was 10.6±4.7. There was no statistically significant association identified between SOFA scores with rise or fall in RDW (p=0.390). The APACHE scores ranged from 12 to 36 with an M±SD of 25.3±5.4. In the rise in RDW group, the M±SD was 25.5±5.1. In the fall in RDW group, the M±SD was 25.2±8.8. There was no statistically significant association identified between APACHE scores with rise or fall in RDW (p=0.834).

A total of 15 patients underwent renal replacement therapy (RRT). Out of these patients, eight patients had a rise in RDW while seven patients had a fall in RDW. There was no statistically significant association identified between the need for RRT and rise or fall in RDW (p=0.766). The hemoglobin values in the study ranged from 12 g/dL to 15.6 g/dL. The M±SD was 13.6±0.9 g/dL. In the rise in the RDW group, the M±SD was 13.8±0.9 g/dL. In the fall in the RDW group, the M±SD was 13.5±0.9 g/dL. There was no statistically significant association between hemoglobin value with rise or fall in RDW (p=0.192). The white blood counts (WBC) values in the study ranged from 12 to 15.6 K/uL. The M±SD WBC values were 17.6±0.9 K/uL, respectively. In the rise in RDW group, the M±SD was 17.2±8.6 K/uL. In the fall in the RDW group, the M±SD was 18.1±9.6 K/uL. There was no statistically significant association between WBC value with rise or fall in RDW (p=0.729).

The CRP values in the study ranged from 17.5 mg/dL to 429.4 mg/dL. The M±SD CRP value was 185.2±107.1 mg/dL. In the rise in the RDW group, the M±SD was 182.5±115.8 mg/dL. In the fall in the RDW group, the M±SD was 187.9±101.6 mg/dL. There was no statistically significant association between CRP value with rise or fall in RDW (p=0.848). The procalcitonin (PCT) values in the study ranged from 1.1 to 100 ng/mL. The M±SD PCT values were 19.6±29.6 ng/mL. In the rise in RDW group, the M±SD was 20.3±30.9 ng/mL. In the fall in RDW group, the M±SD was 19±29.2 ng/mL. There was no statistically significant association between PCT value with rise or fall in RDW (p=0.866).

The serum albumin values in the study ranged from 1.5 g/dL to 3.8 g/dL. The M±SD albumin value was 2.7±0.6 g/dL. In the rise in RDW group, the M±SD was 2.7±0.6 g/dL. In the fall in the RDW group, the M±SD was 2.7±0.5 g/dL. There was no statistically significant association between albumin value with rise or fall in RDW (p=0.811). The serum lactate values in the study ranged from two to 6.8 mmol/L. The M±SD was 3.6±1.1 mmol/L. In the rise in the RDW group, the M±SD was 3.1±0.8 mmol/L. In the fall in the RDW group, the M±SD was 4.2±1.1 mmol/L. There was a statistically significant association between lactate value with rise or fall in RDW (p< 0.05).

The length of stay (LOS) ranged between four to 42 days. The M±SD LOS was 12.2±7.1. In the rise in the RDW group, the M±SD was 12.4±7.9 days. In the fall in RDW group, the M±SD was 12±6.5 days. There was no statistically significant association between LOS with rise or fall in RDW (p=0.846). A total of 17 (28.3%) patients died during this study. In the rise in RDW group, there were 12 (40%) deaths while in the fall in RDW group, there were 5 (16.7%) deaths. There was a statistically significant association found between mortality with rise and fall in RDW (p<0.05). 

The comparison of the various demographic and biochemical parameters in both the groups are summarised in Table 1. 

**Table 1:** The comparison of the various demographic and biochemical parameters in both the groups CCI: Charlson Comorbidity Index, SOFA: Sequential Organ Failure Assessment, APACHE: Acute Physiology And Chronic Health Evaluation, RRT: renal replacement therapy, WBC: white blood counts, CRP: C-reactive protein, PCT: procalcitonin, LOS: length of stay ^a^Expressed as number of patients.

Parameter	Total (n=60)	Rise in RDW (n=30)	Fall in RDW (n=30)	P-value
Male gender^a^	34 (56.7%)	17(56.7%)	17(56.7%)	1
Age (years)	61.7 ± 15.5	61.9 ± 17	61.6 ±14	0.941
CCI score	1.7±1.65	1.7±1.5	1.7±1.8	1
SOFA score	10.1±4	9.7±3.1	10.6±4.7	0.390
APACHE score	25.3±5.4	25.5±5.1	25.2±8.8	0.834
RRT^a^	15	8	7	0.766
Hemoglobin (g/dL)	13.6±0.9	13.8±0.9	13.5±0.9	0.192
WBC (K/uL)	17.6±0.9	17.2±8.6	18.1±9.6	0.729
CRP (mg/dL)	185.2±107.1	182.5±115.8	187.9±101.6	0.848
PCT (ng/mL)	19.6±29.6	20.3±30.9	19±29.2	0.866
Serum albumin (g/dL)	2.7±0.6	2.7±0.6	2.7±0.5	0.811
Lactate (mmol/L)	3.6±1.1	3.1±0.8	4.2±1.1	< 0.05
LOS (days)	12.2±7.1	12.4±7.9	12±6.5	0.846
Mortality^a^	17	12	5	<0.05

**Figure 1:**
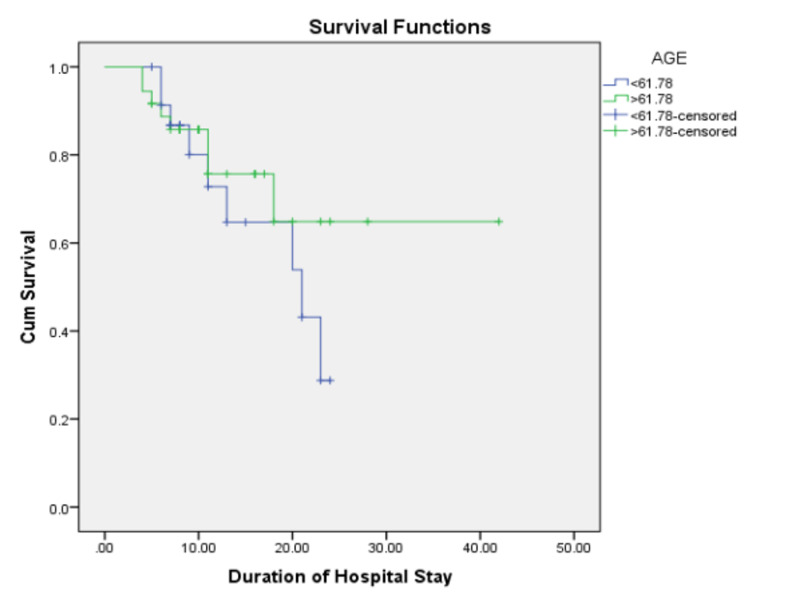
Kaplan-Meier survival analysis curve

A cox multivariate regression analysis demonstrated statistically significant associations between a rise in RDW (p<0.05, HR: 5.6, CI 1.4 to 21.9) and serum hemoglobin < 13.6 g/dL (p<0.05, HR: 3.6, CI 1.1 to 11.4) with increased mortality in this study. A Kaplan-Meier analysis of the rise and fall in RDW with survival trended towards better survival outcomes in the fall in the RDW group but it was not significant (p=0.07) (Figure 1). 

## Discussion

In this prospective comparative observational study, we studied the role of RDW as a prognostic marker of all-cause mortality in patients with sepsis and septic shock presenting to our hospital. A total of 28.3% of patients died during this study. In the rise in RDW group, there were 40% deaths while in the fall in RDW group, there were 16.7% deaths. RDW was significantly associated with the all-cause mortality in septic patients across all age groups and comorbidities in univariate analysis. A rise in RDW and anemia at admission were found to be independent predictors of all-cause mortality in this cohort of septic patients. 

These findings were in contrast to a similar study conducted by Jandial et al. [6]. They looked at a cohort of severely septic patients, and their primary outcome was specified 30-day mortality. They found that although RDW was associated with 30-day mortality in their cohort, it was not found to be an independent predictor of 30-day mortality. Furthermore, they found significant associations between the APACHE II scores, PaO2/FiO2 ratio, serum albumin, and serum fibrinogen to be independent predictors of 30-day mortality. In our study, these findings could not be corroborated, although we did not study fibrinogen values. In contrast, another study by Kim et al. looking at the prognostic value of changes in RDW in patients receiving a standardized resuscitation algorithm for severe sepsis and shock found that a change in RDW value during the initial 72 hours following admission, had a significant impact on the all-cause mortality [7]. They found that a rise in RDW from baseline in the initial 72 hours after hospitalization, was a strong and independent predictor of mortality in septic patients even after adjusting for confounders. They concluded that RDW was a dynamic risk marker in septic patients and that a rise in RDW could serve as an early indicator of adverse outcomes including mortality [9]. The highlight of this study was that even 90-day mortality was significantly impacted by a rise in the RDW values. These findings closely resembled the findings of this study. 

Jo et al. reported a similar finding of an association between baseline RDW values and mortality in a retrospective study involving severe sepsis patients [9]. However, they concentrated only on baseline values and did not perform trend analysis. They found that the RDW of non-survivors was significantly higher than the mean RDW values of survivors with severe sepsis and shock. They further found that there was a graded relationship between RDW and 28-day mortality. RDW also had an association with the severity of the sepsis and was also an independent prognostic marker of 28-day mortality in their cohort of severely septic patients. Our prospective study enabled us to corroborate these results, along with an opportunity to study the impact of serial RDW rise or fall in a similar cohort of patients. We were able to demonstrate similar results, further adding evidence to the role of RDW as a potential, widely available, and relatively cost-effective prognostic marker in the management of sepsis and septic shock.

Even though the mechanism causing the association between a rise in RDW and mortality in septic patients is not yet completely understood, numerous hypotheses have been suggested in previous literature. Systemic inflammation can predict progressive illness, cardiovascular death, and overall mortality in ICU patients. Response to systemic inflammation may impact bone marrow function and subsequently the iron metabolism [13,14]. Pro-inflammatory cytokines inhibit erythropoietin-induced erythrocyte proliferation and maturation, which are associated with RDW increases [15]. High oxidative stress has also been implicated in the association between RDW and mortality through the generation of reactive oxygen species by activated leukocytes. This induces a rise in RDW by virtue of the reduction of RBC survival and subsequent increase in the release of large premature RBCs into the peripheral circulation [16]. 

This study did not find any associations between routinely used pro-inflammatory markers such as CRP or PCT with a change in RDW levels. Although studies looking at the relationship between RDW and other pro-inflammatory markers were lacking in published literature in a septic cohort, other studies in other acute illnesses have previously demonstrated significant associations between higher inflammatory markers and higher RDW values [17]. Although numerous studies have looked at the role of RDW in inflammation and adverse clinical outcomes in sepsis, the temporal relationship of RDW and other pro-inflammatory markers is not well understood and could warrant further research. Our study also did not find significant correlations between the commonly used scores to prognosticate sepsis, such as the SOFA or APACHE II score. This could very well have been the result of a relatively low sample size. 

There are several limitations to our study. We arbitrarily determined serial RDW value determination at 72 hours after admission as the second measurement. Whether changes in RDW during the initial 72 hours can accurately predict the pathophysiologic changes in critically ill patients is not clear. We did not investigate vitamin B12 deficiency or the reticulocyte count, which could confound the affected RDW values and, thus, might have limited the interpretation of study results. The relatively low sample size and the number of events were not large enough to establish the statistical significance of increased risk among categorized groups. Therefore, a larger multicenter study with serial RDW measurements may be warranted to further clarify the predictive value of changes in RDW.

## Conclusions

We found that an increase in RDW from baseline during the initial 72 hours after admission is significantly associated with adverse clinical outcomes including mortality. The mortality in the rise in the RDW group, as well as overall mortality, were significantly higher than the mortality in the reduced RDW group. Hence, serial RDW measurements could be used as a prognostic factor in severe sepsis and septic shock.

A combination of baseline RDW value and demonstration of a rise in RDW could be a promising independent prognostic marker of mortality in patients with sepsis or septic shock. Further research is warranted to determine the exact mechanisms underlying this association between RDW and mortality. This study supports the need for future investigations concerning changes in RDW and the stratification of critically ill patients at risk for mortality based on the RDW values.
